# Integrin αEβ7 is involved in the intestinal barrier injury of sepsis

**DOI:** 10.18632/aging.203839

**Published:** 2022-01-18

**Authors:** Jia-Kui Sun, Qian Zhang, Xiao Shen, Jing Zhou, Xiang Wang, Su-Ming Zhou, Xin-Wei Mu

**Affiliations:** 1 Department of Geriatrics Intensive Care Unit, The First Affiliated Hospital of Nanjing Medical University (Jiangsu Province People’s Hospital), Nanjing 210029, Jiangsu Province, China; 2 Department of Critical Care Medicine, Nanjing First Hospital, Nanjing Medical University, Nanjing 210006, Jiangsu Province, China

**Keywords:** integrin, αEβ7, E-cadherin, Th9 cells, intestinal mucosal barrier

## Abstract

Background: IL-9-producing CD4(+) T (Th9) cell was related to acute intestinal barrier injury in sepsis. Integrin αEβ7 was an important lymphocyte homing receptor on the surface of intestinal Th9 cells. However, the roles of αEβ7 in the intestinal injury caused by Th9 cells were not clear in sepsis.

Methods: To investigate the roles of αEβ7 in the intestinal injury caused by Th9 cells in sepsis model, the Th9 cells percentages, αEβ7, E-cadherin, IL-9, and D-lactate levels in both serum and intestinal tissue were measured. The intestinal histopathology, epithelium apoptosis, and mucosal permeability measurement were also performed. The survival rate of septic rats was recorded daily for 14 days.

Results: Rats were assigned to four cohorts: control cohort, sepsis cohort, sepsis+αEβ7i (αEβ7 inhibition) cohort, and sepsis+αEβ7e (αEβ7 overexpression) cohort. The Th9 cells percentages, αEβ7, IL-9, and D-lactate levels of the sepsis cohort were significantly higher than those of the control cohort. The levels of these variables were also elevated progressively in the sepsis+αEβ7i cohort, sepsis cohort, and sepsis+αEβ7e cohort. The E-cadherin levels were decreased progressively in the control cohort, sepsis+αEβ7i cohort, sepsis cohort, and sepsis+αEβ7e cohort. Moreover, αEβ7 overexpression could decrease the 14-day survival rate. The findings of histopathology staining, apoptosis detection, and intestinal permeability test also confirmed that the barrier injury was deteriorated or relieved by elevating or decreasing the αEβ7 expression levels, respectively.

Conclusions: Integrin αEβ7 was closely associated with the intestinal barrier injury caused by Th9 lymphocytes in sepsis.

## INTRODUCTION

Sepsis is a potentially fatal disease characterized by organ dysfunction as a result of an improperly controlled host response to pathogens [1, 2]. Intestinal tract is considered as one of the most vulnerable organs to sepsis [3, 4]. After sepsis, intestinal mucosal permeability increased, resulting in the occurrence and progression of secondary infection [3–5]. Hence, gastrointestinal dysfunction is a crucial factor leading to poor prognosis in critically ill patients [6, 7]. Nevertheless, the exact mechanism that regulates the injury to the intestinal barrier in sepsis has not been undetermined. Our previous investigations revealed that T helper lymphocytes were involved in the intestinal barrier disorder in sepsis [8, 9]. Besides the regular T regulatory (Treg) lymphocytes, T helper (Th) 1, Th2 and Th17, IL-9-producing CD4+ T helper (Th9) cells was also confirmed to cause tumor, infection, or inflammatory bowel disease [10–13].

It is predominantly through the integrated activation of interleukin-4 (IL-4) and transforming growth factor-beta (TGF-β) that Th9 cells are primarily generated and differentiated from naive T cells, which preferentially secrete IL-9 and IL-10 [11, 12]. A study by Nalleweg et al. [14] showed that the expression of interleukin-9 receptor was substantially elevated in gut epithelial cells and that IL-9 was involved in the development of ulcerative colitis. Our recent research also determined that the proportions of Th9 cells, as well as the expression levels of IL-9, were particularly associated with acute intestinal barrier dysfunction in sepsis [15]. Therefore, IL-9 and Th9 cells may be possible targets for the treatment of gastrointestinal dysfunction in early sepsis. Zundler et al. [10] reported that αEβ7 integrin were highly expressed on CD4+ Th9 cells both in the peripheral blood and the intestine of mice. Th9 cells could homing into the intestinal epithelial layer and lamina propria through the combination of αEβ7 and its ligand E-cadherin, and then lead to the local inflammation of intestine [16, 17]. Blockade of αEβ7 suppressed the aggregation of Th9 lymphocytes and CD8+ cells in the inflamed gut [10]. These results indicated that blockade of αEβ7 may be a useful treatment for the intestinal barrier damage of sepsis. Accordingly, the objective of the present research was to examine the roles of αEβ7 in the intestinal injury caused by Th9 lymphocytes in sepsis model.

## MATERIALS AND METHODS

### Sepsis model and cohorts

The animal tests were performed for the purpose of determining the specific function of αEβ7 in the intestinal barrier dysfunction associated with sepsis. Zhejiang Academy of Medical Sciences' animal facility (Zhejiang, China) provided adult SD rats aged between 6-8 weeks and weighing between 180-220g. As described in our previous study, sepsis was stimulated by means of the commonly used procedure of cecum ligation and puncture (CLP) [15]. The rats were anesthetized using 10 percent chloral hydrate (0.3 ml/100 g) injected intraperitoneally. Under sterile condition, a 2.5 cm incision was made from the middle abdomen of rats. Following the incision, ligation of the cecum was performed at the middle section using 3-0 silk, followed by piercing using a 20-gauge needle. Subsequently, the cecum was surgically implanted into the abdominal cavity. The same laparotomy procedure was carried out on sham-operated (control) rats who did not have their cecum ligatured or punctured. Hypotension was prevented by administering antibiotics and normal saline.

The rats were divided into 4 cohorts (each with n = 12 rats) using a randomization procedure, namely control cohort, sepsis cohort, sepsis+αEβ7i (αEβ7inhibition) cohort, and sepsis+αEβ7e (αEβ7 overexpression) cohort. Adeno-associated virus (AAV) of αEβ7 interference was administered to the sepsis+αEβ7i cohort rats by intraperitoneal injection, whereas AAV of αEβ7 overexpression was administered to the sepsis+αEβ7e cohort rats. The production of AAV referred to our previous research [15, 18]. Briefly, cDNA clone fragment encoding αEβ7 or αEβ7 shRNA cassette were transferred into rAAV plasmid, respectively (Genepharma, Shanghai, China). After co-transfection with AAV package, the plasmid was shuttled into AAV-293 cell line. Following the precipitation of ammonium sulfate as well as iodixanol continuous gradient centrifugation procedures, we collected the AAV viral particle and performed the real-time polymerase chain reaction to determine the genome titers of the virus. The dosage and titers of AAV particle were 100 μl and 2 × 10^11^ vg/ml, respectively. After 7 days of AAV injection, sepsis was induced.

Sacrificing of the rats was performed on the seventh day following the modeling of sepsis. After cardiac puncture, blood was collected with heparin tube and centrifuged (10 min, 1500×g). The colon was taken from abdominal cavity, and then the tissue was sliced into about 4 mm slices. We then embedded the sample in paraffin, followed by fixing for 12 to 24 hours in 4 percent buffered paraformaldehyde.

### Cytokine and flow cytometry measurement

Flow cytometry was utilized for the purpose of determining the percentage of Th9 cells present in intestinal tissue or serum [Donkey anti-sheep antibody Alexa fluor647 (Abcam, ab150179), FITC-CD4 antibody (eBioscience, 11-0400-82), IL-9 antibody (eBioscience, PA5-47584)]. The enzyme-linked immunosorbent assay (ELISA) kits were utilized for the detection of the serum levels of αEβ7 (MyBioSource, San Diego, CA, USA), E-cadherin (Cusabio, Wuhan, China), IL-9 (Cusabio, Wuhan, China), as well as D-lactate (a barrier function biomarker) (MyBioSource, San Diego, CA, USA).

### Western blot

After rat sacrifice, the intestinal sample was disintegrated in radio immunoprecipitation assay lysis buffer. Following the lysate isolation in sodium dodecyl sulfate-polyacrylamide gel electrophoresis, the lysate was loaded onto a nitrocellulose membrane. Using a bicinchoninic acid kit (Bio-Rad Laboratories, Hercules, CA, USA), we determined the expression level of protein in the lysate sample. The lysate blotting was subjected to incubation using each antibody as follows: GAPDH antibody (ZEN-bio, diluted in 1:5000), HRP-labeled anti-mouse antibody (Beyotime, Shanghai, China), D-lactate antibody (Abcam, diluted in 1:1000), E-cadherin antibody (Abcam, diluted in 1:1000), αEβ7 antibody (affinity, diluted in 1:1000), IL-9 antibody (Abcam, diluted in 1:1000), and horseradish peroxidase (HRP)-labeled anti-rabbit antibody (Beyotime, Shanghai, China). Tanon-enhanced chemiluminescence substrate of the Tanon 5200 imaging system was used (Tanon, Shanghai, China) to produce the images.

### Histopathology

After the rats had been sacrificed, the intestinal sample was removed and subjected to preservation with 4 percent formalin, followed by embedding. Hematoxylin and eosin (H&E) were utilized for the purpose of staining the tissue slices that were cut into 4-micron thicknesses. Subsequently, a Nikon H550S microscope and a Nikon DS-Ri2 imaging system (Nikon, Japan) were utilized to visualize and evaluate the sections. The Chiu score was applied to evaluate the extent of intestinal damage.

### Apoptosis

We employed the terminal deoxynucleotidyl transferase (TUNEL) assay to investigate if intestinal mucosal epithelial cells had undergone apoptotic cell death. A 4 percent buffered paraformaldehyde solution was utilized for the purpose of fixing the tissue, followed by embedding and slicing into 4 micron-thick slices. A TUNEL kit (KeyGenBiotech, Nanjing, China) was then used to stain the sections following this procedure. With the aid of an optical microscope, each sample was identified, and the number of cells was enumerated in 4 fields chosen at random. Cells with brown nuclei were considered to be positive apoptotic cells.

### Intestinal permeability

The fluorescence tracing method was performed to observe the intestinal mucosa permeability of rats. A 15 cm long intestinal segment was subjected to ligation at one end, followed by the gradual injection of a tracer (EZ-Link NHS-Biotin, 2 mg/ml) (Thermo Fisher Scientific, MA, USA) into the distal intestine. Subsequently, a 5 cm long intestine piece was chosen, followed by fixing in 4 percent paraformaldehyde for 3 hours, after which it was rinsed 3 times using phosphate buffer solution and sliced. The samples were subjected to incubation for 30 min at ambient temperature with streptavidin. The tracer distribution in intestine was measured by laser confocal microscope.

### Analysis of survival rate

Using a randomization method, the rats were classified into 4 cohorts, namely control cohort, sepsis cohort, sepsis+αEβ7i cohort, and sepsis+αEβ7e cohort (n =10 in each cohort). After being afflicted with sepsis, the rats were monitored on a daily basis for two weeks for the purpose of determining their survival rate.

### Statistical analysis

First, the Kolmogorov-Smirnov test was employed to evaluate if the data were normally distributed. Data that lacked normal distribution were presented as the medians (interquartile ranges) and comparisons for these data were performed utilizing the Kruskal-Wallis or Mann-Whitney *U* test. The data with normal distribution were presented as the means ± standard deviation and were analyzed utilizing *t*-tests. Categorical variables were presented as percentages or absolute numbers, and they were subjected to a comparison using the *χ^2^* test or Fisher's exact test, as applicable. For the multiple testing of the general linear model, the analysis of variance (ANOVA), as well as the least significant difference (LSD) post-hoc tests, were conducted. The Kaplan-Meier technique was utilized to generate survival curves for 14 days following the induction of sepsis, and the results were evaluated using the log-rank test. For the purpose of performing data analysis, IBM Statistical Package for the Social Sciences (SPSS, version: 22.0, New York, USA) software was utilized and two-sided *P* <0.05 was confirmed to be the threshold of statistical significance.

## RESULTS

**Figure 1:**
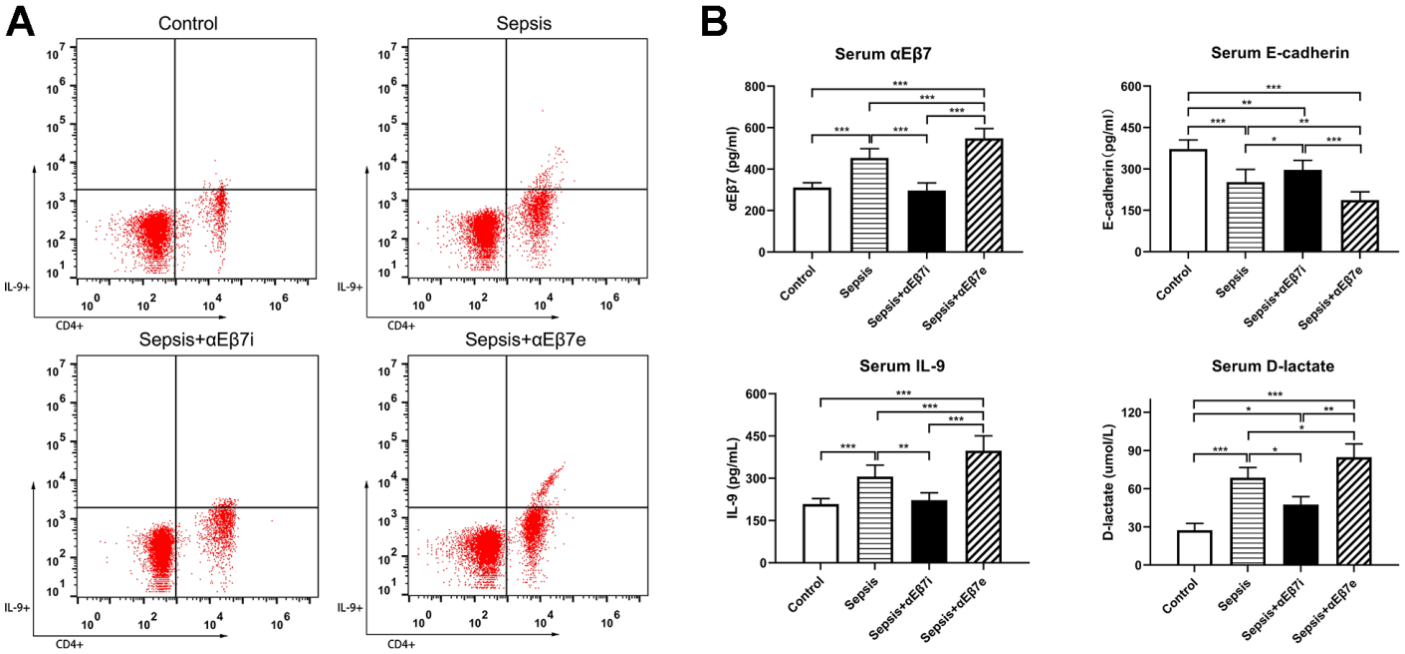
(**A**) The serum percentages of IL-9-producing CD4(+) T cells of rats in control cohort (0.45%±0.04%), sepsis cohort (1.63%±0.46%), sepsis+αEβ7i cohort (0.93%±0.26%), and sepsis+αEβ7e cohort (2.98%±0.64%), respectively (*P* <0.05). The right upper quadrant of each figure represents a subset of IL-9-producing CD4(+) T cells. (**B**) The serum αEβ7, E-cadherin, IL-9, and D-lactate levels among the four cohorts. (*P* <0.01). * *P* <0.05, ** *P* <0.01, *** *P* <0.001.

A sum of 48 SD rats was classified at random to the 4 cohorts, namely control cohort, sepsis cohort, sepsis+αEβ7i cohort, and sepsis+αEβ7e cohort. As demonstrated in Figure 1A, the serum Th9 cells proportions of the control cohort were relatively lower than those of the sepsis cohort (0.45% ± 0.04% vs. 1.63% ± 0.46%, *P* =0.001) or the sepsis+αEβ7e cohort (0.45% ± 0.04% vs. 2.98% ± 0.64%, *P* <0.001). Moreover, the serum Th9 cells proportions of the sepsis+αEβ7e cohort were shown to be elevated in contrast with those of the sepsis cohort (2.98% ± 0.64% vs. 1.63% ± 0.46%, *P* <0.001) or the sepsis+αEβ7i cohort (2.98% ± 0.64% vs. 0.93% ± 0.26%, *P* <0.001). The serum Th9 cell proportions of the sepsis cohort were also elevated in contrast with those of the sepsis+αEβ7i cohort (1.63% ± 0.46% vs. 0.93% ± 0.26%, *P* =0.027).

The serum concentrations of αEβ7, IL-9, and D-lactate were reduced in the control cohort in contrast with the sepsis cohort and the sepsis+αEβ7e cohort (Figure 1B, *P <*0.001*)*. The serum concentrations of the three variables were progressively elevated in the sepsis+αEβ7i cohort, sepsis cohort, and sepsis+αEβ7e cohort (Figure 1B, *P <*0.05*)*. The serum E-cadherin levels were progressively reduced in the control cohort, sepsis+αEβ7i cohort, sepsis cohort, and sepsis+αEβ7e cohort (Figure 1B, *P <*0.05*)*.

**Figure 2:**
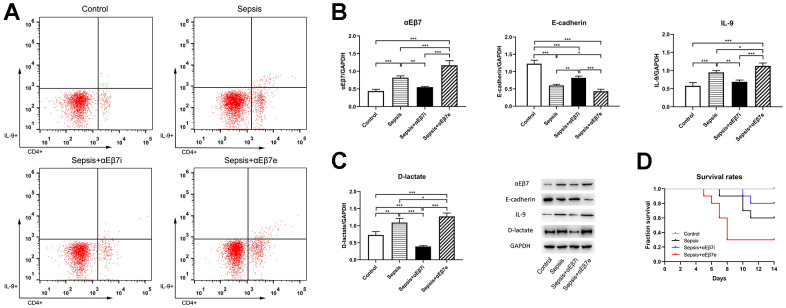
(**A**) The intestinal tissue percentages of IL-9-producing CD4(+) T cells of rats in control cohort (0.28%±0.06%), sepsis cohort (1.05%±0.05%), sepsis+αEβ7i cohort (0.33%±0.03%), and sepsis+αEβ7e cohort (1.41%±0.05%), respectively (*P* <0.05). The right upper quadrant of each figure represents a subset of IL-9-producing CD4(+) T cells. (**B**, **C**) The expression levels of αEβ7, E-cadherin, IL-9, and D-lactate in intestinal tissue of rats among the four cohorts. (**D**) Survival curves for up to 14 days of rats among the four cohorts (*P* <0.05). * *P* <0.05, ** *P* <0.01, *** *P* <0.001.

The differences of Th9 cells proportions, αEβ7, E-cadherin, IL-9, and D-lactate concentrations in intestine were shown in Figure 2. As presented in Figure 2A, the tissue Th9 cells proportions of the control cohort were found to be reduced as opposed to those of the sepsis cohort (0.28% ± 0.06% vs. 1.05% ± 0.05%, *P* <0.001) or the sepsis+αEβ7e cohort (0.28% ± 0.06% vs. 1.41% ± 0.05%, *P* <0.001). Moreover, the tissue Th9 cells proportions were progressively elevated in the sepsis+αEβ7i cohort (0.33% ± 0.03%), sepsis cohort, and sepsis+αEβ7e cohort (Figure 2A, *P <*0.01*)*. The results of western blot (Figure 2B, 2C) revealed that the levels of αEβ7, IL-9, and D-lactate expression in the intestine were relatively low in the control cohort in contrast with the those in the sepsis cohort (*P* <0.01) or the sepsis+αEβ7e cohort (*P* <0.001). It was also revealed that the expression levels of the three variables in intestinal tissue were progressively elevated in the sepsis+αEβ7i cohort, sepsis cohort, and sepsis+αEβ7e cohort (Figure 2B, 2C, *P <*0.05*)*. The E-cadherin levels in intestinal tissue were progressively reduced in the control cohort, sepsis+αEβ7i cohort, sepsis cohort, and sepsis+αEβ7e cohort (Figure 2B, 2C, *P <*0.01*)*.

As indicated in Figure 2D, the survival likelihood of rats in the sepsis+αEβ7e cohort (30.0%) was considerably reduced as opposed to that in the other three cohorts (*P* <0.05). The survival likelihood of rats in the sepsis cohort (60.0%) was also relatively reduced in contrast with that in the control cohort (*P* =0.03). No difference was found between the sepsis+αEβ7i cohort (80.0%) and the control cohort (100%) (*P* >0.05) or the sepsis cohort (60.0%) (*P* >0.05).

**Figure 3:**
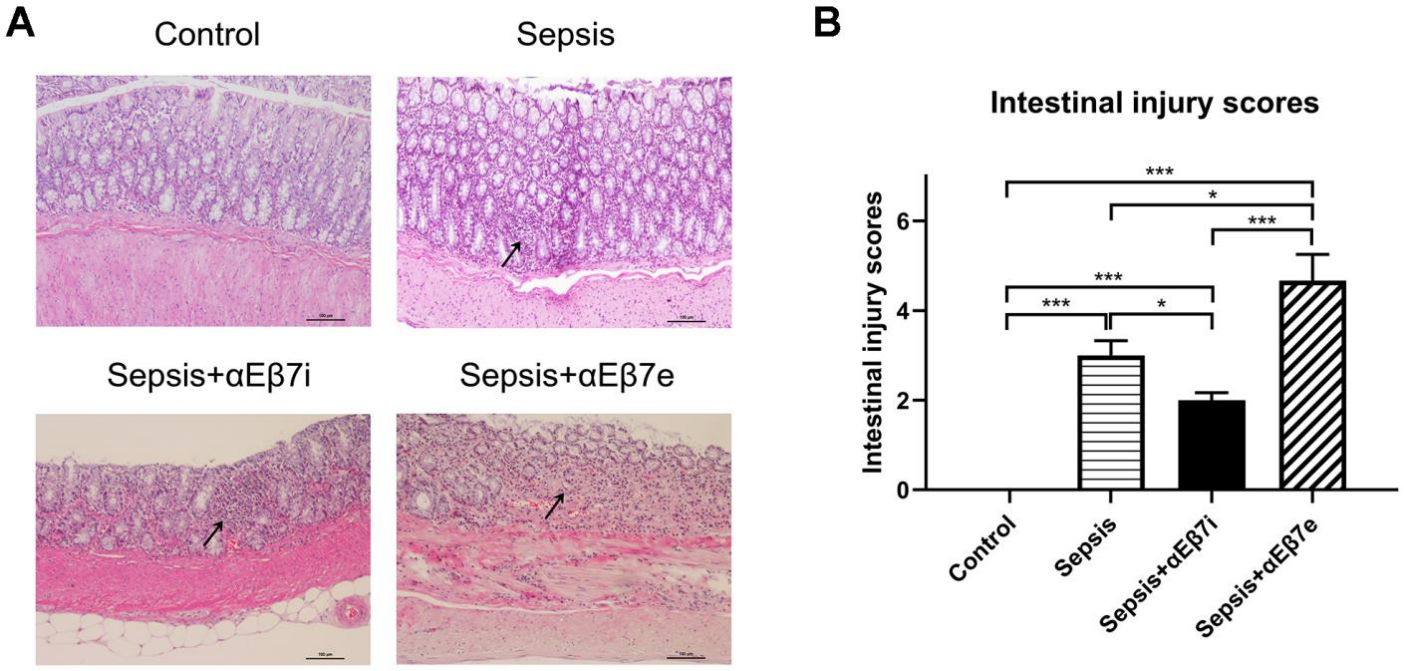
(**A**) The hematoxylin and eosin stain of intestinal mucosa in rats of control cohort, sepsis cohort, sepsis+αEβ7i cohort, and sepsis+αEβ7e cohort, respectively. (**B**) The intestinal injury scores of rats among the four cohorts. * *P* <0.05, ** *P* <0.01, *** *P* <0.001.

**Figure 4:**
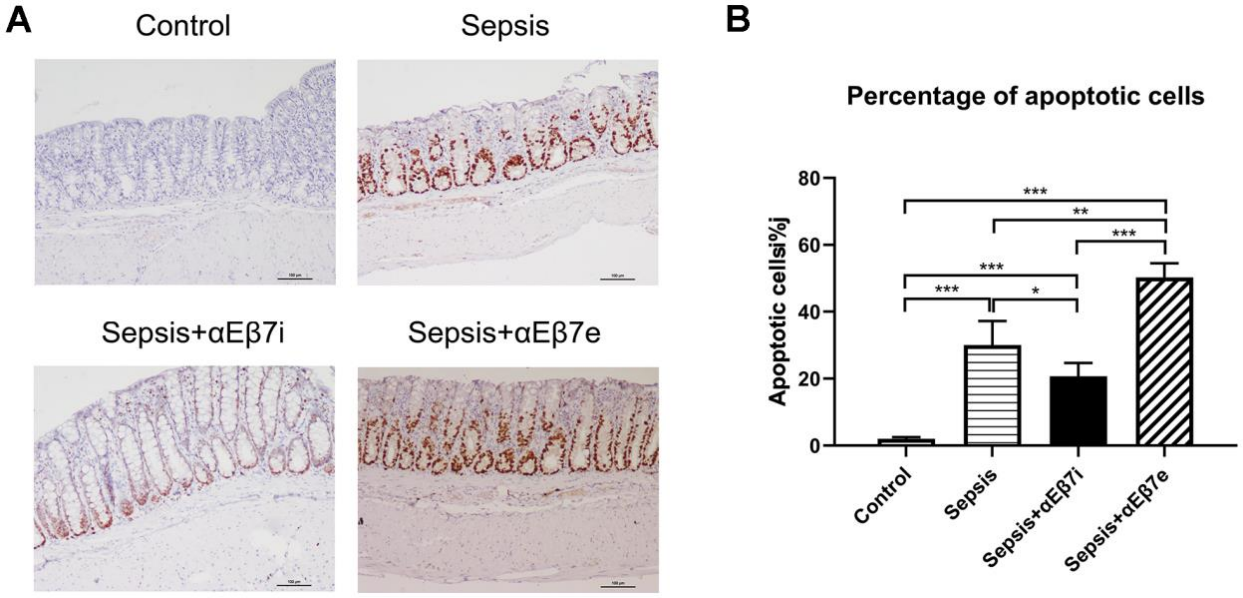
(**A**) The epithelial cells apoptosis detection of intestinal mucosa in rats of control cohort, sepsis cohort, sepsis+αEβ7i cohort, and sepsis+αEβ7e cohort, respectively. (**B**) The percentages of apoptotic cells of intestinal mucosa in rats among the four cohorts. * *P* <0.05, ** *P* <0.01, *** *P* <0.001.

HE staining results were shown in Figure 3. The intestinal epithelium was intact and mucosa was normal without hemorrhage in the non-septic controls (Figure 3A). The inflammatory cell infiltration or lamina propria hemorrhage was found in the sepsis cohort (Figure 3A). The intestinal mucosal damage was mitigated or enhanced by αEβ7 interference or αEβ7 overexpression, respectively (Figure 3A). The morphological injury was measured by Chiu’s score (Figure 3B). The Chiu’s scores were increased progressively in the control cohort, sepsis+αEβ7i cohort, sepsis cohort, and sepsis+αEβ7e cohort (Figure 3B, *P <*0.05*)*. The findings of TUNEL apoptosis detection were illustrated in Figure 4A. Brown nuclei were observed in the intestinal mucosa, indicating that the cells were apoptotic. As shown in Figure 4B, the percentages of apoptotic cells were also increased progressively in the control cohort, sepsis+αEβ7i cohort, sepsis cohort, and sepsis+αEβ7e cohort (*P* <0.01).

**Figure 5:**
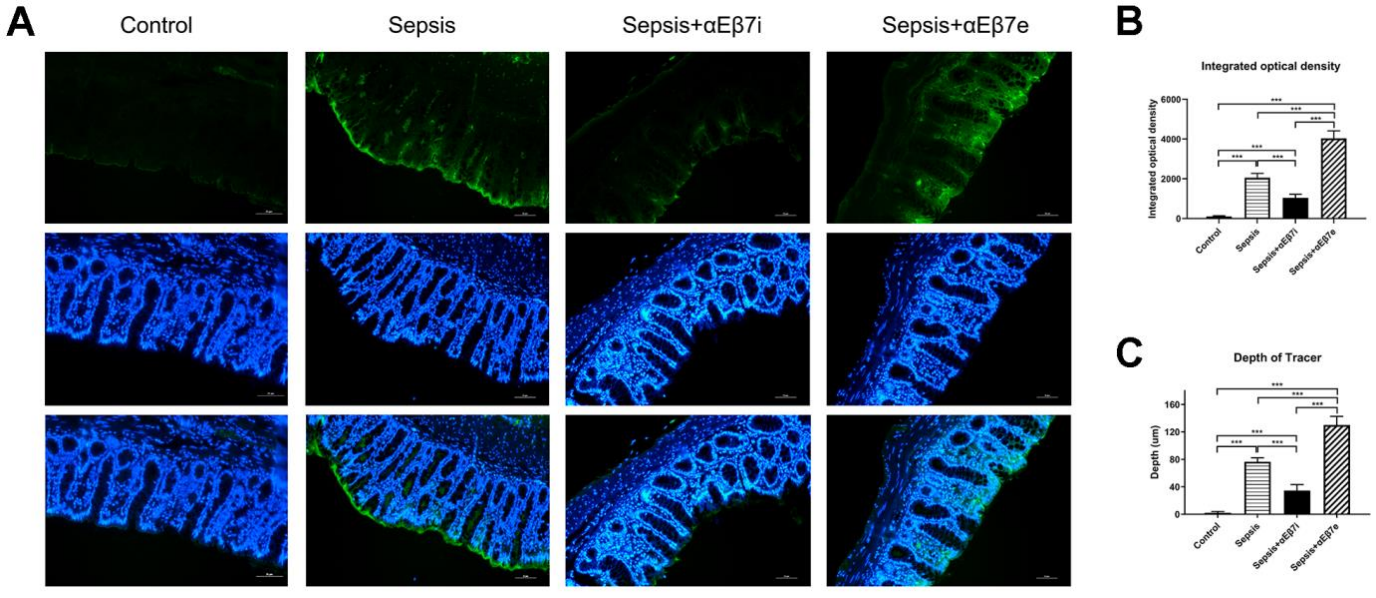
(**A**) Detection of intestinal mucosal permeability in rats of control cohort, sepsis cohort, sepsis+αEβ7i cohort, and sepsis+αEβ7e cohort, respectively. (**B**) The integrated optical density (IOD) of fluorescent staining in the intestinal mucosa of rats among the four cohorts. (**C**) The penetration depth of tracer in the intestinal mucosa of rats among the four cohorts. * *P* <0.05, ** *P* <0.01, *** *P* <0.001.

The fluorescence tracing method was performed to observe the intestinal mucosa permeability (Figure 5). The fluorescent tracer was green. The tracer distribution was only detected on the surface of intestinal mucosa in the control cohort (Figure 5A). The tracer was distributed into the intestinal muscular layer in the sepsis cohort (Figure 5A). After αEβ7 overexpression, the tracer was observed in the intestinal submucosal layer, whereas after αEβ7 interference, the tracer was only observed in the mucosa epithelial layer (Figure 5A). As shown in Figure 5B, the integrated optical density (IOD) of tracer were elevated progressively in the control cohort, sepsis+αEβ7i cohort, sepsis cohort, and sepsis+αEβ7e cohort (*P* <0.05). Furthermore, the depth of tracer penetration was also elevated progressively in the control cohort, sepsis+αEβ7i cohort, sepsis cohort, and sepsis+αEβ7e cohort (Figure 5C, *P* <0.001).

## DISCUSSION

Gastrointestinal dysfunction or intestinal barrier injury is closely related to poor prognosis of critically ill patients [6]. The underlying mechanisms of intestinal barrier injury in sepsis have not been clarified. Besides the regular T helper lymphocytes, Th9 cells was found to be involved in the acute intestinal damage of sepsis through the IL-9 pathway in our previous study [15]. However, it is not clear how the Th9 cells are regulated in sepsis. In this study, we confirmed the important roles of αEβ7 in the intestinal injury caused by Th9 lymphocytes in sepsis. The Th9 cell proportions, αEβ7, IL-9, and D-lactate levels in both serum and intestinal tissue of the sepsis cohort were higher in contrast with those of the control cohort. The levels of these markers in both serum and intestinal tissue were also elevated progressively in the sepsis+αEβ7i cohort, sepsis cohort, and sepsis+αEβ7e cohort. The E-cadherin levels in both serum and intestinal tissue were reduced progressively in the control cohort, sepsis+αEβ7i cohort, sepsis cohort, and sepsis+αEβ7e cohort. Survival rate analysis indicated that αEβ7 overexpression reduced the 14-day survival likelihood of septic rats. The findings of HE staining, TUNEL apoptosis detection, and intestinal permeability test also suggested that the intestinal damage was deteriorated or relieved by elevating or decreasing the αEβ7 expression levels, respectively.

Integrins are transmembrane molecules that mediate the interactions between cells and extracellular matrix [19]. They are specific heterodimers consisted of an α and β subunit, each passing through the plasma membrane [19]. Integrin αEβ7 (CD103) was confirmed to be an important lymphocyte homing receptor on the surface of intestinal Th9 cells in inflammatory bowel diseases [10, 17, 19, 20]. E-cadherin is the only known ligand of αEβ7 at present [19]. Th9 cells can be homed to the intestinal epithelial layer and lamina propria through the combination of αEβ7 and E-cadherin [10, 17, 19]. However, the exact roles of αEβ7 in the intestinal barrier damage owing to Th9 lymphocytes in sepsis were not determined. This pre-clinical research firstly confirmed that αEβ7 was closely correlated with the intestinal damage in sepsis. Our results also suggested that blockade of αEβ7 may be beneficial to the treatments of gastrointestinal injury with expansion of Th9 cells in sepsis.

E-cadherin is a main cadherin produced in the colonic crypt epithelium [19, 21]. The colon consists of an epithelial monolayer that contained an E-cadherin-dependent barrier, which is essential for the organ homeostasis [21]. Doshi A et al. reported that the levels of IL-9 expression were negatively related with the levels of membrane bound E-cadherin in eosinophilic esophagitis [22]. Reports on intestinal E-cadherin expression were inconsistent in inflammatory bowel diseases, but most results advocated a decreased expression [10, 17, 19]. Our study found that the E-cadherin levels in both serum and intestinal tissue were reduced progressively in the control group, sepsis+αEβ7i cohort, sepsis cohort, and sepsis+αEβ7e cohort, whereas the IL-9 or αEβ7 were opposite. This phenomenon showed that the αEβ7 expression of Th9 cells was negatively correlated with the E-cadherin expression of colonic epithelium in sepsis. Furthermore, IL-9 and E-cadherin may exert a feedback relation via underlying pathways, although the exact mechanisms were not recognized. The differences in the proportions of Th9 cells or the levels of IL-9 were consistent with the findings of our previous study [15].

The intestine is often regarded as a vital immunological organ of the body. In the case of sepsis, a vicious loop of acute intestine damage and systemic dysregulated infection occurs [4–6]. We previously discovered that T lymphocytes, especially Th9 cells, were strongly associated with the acute intestinal malfunction and immunological disorder in sepsis, as evidenced by our prior research [8, 9, 15]. In this research, we confirmed that the overexpression of αEβ7 on the surface of Th9 cells increased epithelial cell apoptosis, inflammatory cell infiltration, and mucosal permeability of intestine. Moreover, αEβ7 overexpression could decrease the 14-day survival rate. D-lactate is a commonly used indicator of intestinal barrier function. The levels of D-lactate in both intestinal tissue and serum were elevated progressively in the sepsis+αEβ7i cohort, sepsis cohort, and sepsis+αEβ7e cohort. The results were also consistent with the findings of our previous study [15].

There are certain limitations to the present pre-clinical trial that should be taken into consideration seriously. Owing to the limited sample size used in the present research, the findings may be inconclusive; thus, large-scale studies need to be conducted for the purpose of confirming the validity of our findings. Furthermore, because the findings of this study were only based on the animal sepsis model, the actual expression of αEβ7 in patients with sepsis should be tested by further clinical studies.

## CONCLUSIONS

This study indicated that αEβ7 integrin was closely associated with the intestinal barrier injury caused by Th9 lymphocytes in sepsis. A blockade of αEβ7 may alleviate the barrier injury in sepsis.
